# Use of re-randomized data in meta-analysis

**DOI:** 10.1186/1471-2288-5-17

**Published:** 2005-05-10

**Authors:** Iztok Hozo, Benjamin Djulbegovic, Otavio Clark, Gary H Lyman

**Affiliations:** 1 Indiana University Northwest, Department of Mathematics, Gary, IN, USA; 2 Interdisciplinary Oncology Program, H. Lee Moffitt Cancer Center and Research Institute at the University of South Florida, Tampa, FL, USA; 3 Instituto do Radium de Campinas, Av Heitor Penteado 1780, Campinas-SP, Brasil; 4 James P. Wilmot Cancer Center, University of Rochester Medical Center, Rochester, NY, USA

## Abstract

**Background:**

Outcomes collected in randomized clinical trials are observations of random variables that should be independent and identically distributed. However, in some trials, the patients are randomized more than once thus violating both of these assumptions. The probability of an event is not always the same when a patient is re-randomized; there is probably a non-zero covariance coming from observations on the same patient. This is of particular importance to the meta-analysts.

**Methods:**

We developed a method to estimate the relative error in the risk differences with and without re-randomization of the patients. The relative error can be estimated by an expression depending on the percentage of the patients who were re-randomized, multipliers (how many times more likely it is to repeat an event) for the probability of reoccurrences, and the ratio of the total events reported and the initial number of patients entering the trial.

**Results:**

We illustrate our methods using two randomized trials testing growth factors in febrile neutropenia. We showed that under some circumstances the relative error of taking into account re-randomized patients was sufficiently small to allow using the results in the meta-analysis. Our findings indicate that if the study in question is of similar size to other studies included in the meta-analysis, the error introduced by re-randomization will only minimally affect meta-analytic summary point estimate.

We also show that in our model the risk ratio remains constant during the re-randomization, and therefore, if a meta-analyst is concerned about the effect of re-randomization on the meta-analysis, one way to sidestep the issue and still obtain reliable results is to use risk ratio as the measure of interest.

**Conclusion:**

Our method should be helpful in the understanding of the results of clinical trials and particularly helpful to the meta-analysts to assess if re-randomized patient data can be used in their analyses.

## Background

Statistical tests performed to evaluate differences in the outcomes collected in randomized clinical trials (RCTs) are based on the assumption that all the data come from random variables that are independent of each other. However, in some trials, the patients are randomized more than once, usually twice. Investigators often pool these events and report data for all randomized patient episodes instead of episode per patient. Therefore, the independence assumption can be violated.

Concerns about the re-randomization of patients on febrile neutropenia trials have been raised by some authors [1] due to a possible violation of the principle of independency. However, this practice is accepted by the Immunocompromised host society as a valid one [2] and has been used in some of the trials performed in neutropenic patients.

We have recently encountered this problem during our conduct of meta-analysis (MA) on the role of colony-stimulating factors in the treatment of febrile neutropenia [3] where two out of the 12 trials reported analysis according to febrile neutropenia episodes instead of episodes per patients. In this paper, we address the issue whether the combined (pooled) risk difference (RD) after re-randomization is considerably different from the RD if the patients were not re-randomized.

## Methods

### Assumptions

Assuming that once an event (FN episode) occurred in a patient the probability of another occurrence is likely to be different from its initial value, we introduce a parameter *d *measuring this change:



Previously, Chouaid et al. [4] provided the data on the probability of FN after each randomization. Data were available from 39 patients, of which seven had an event; six of these patients were re-randomized and four of them had another occurrence of event.

Thus, accordingly an estimate for *d *can be obtained by . To avoid messy notation, we will omit the 'hat' from the estimates of these parameters. This indicates that the patient who develops an FN episode after first cycle will have about 3.71 times higher likelihood of development of FN in the second chemotherapy cycle. Note that our calculations also work for values *d *< 1.

On the other hand, the patients who did not experience an episode of FN are usually less likely to experience it after re-randomization. The probability of an FN episode in a patient who didn't experience an episode of FN in the first round is *c *times smaller (or larger, if *c > *1) in the second round of randomization. In this example, .

**Figure 1:**
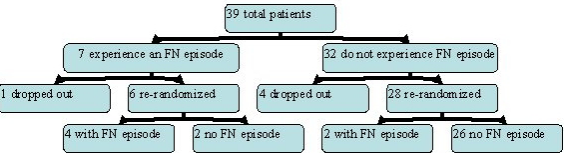
Chouaid et al. study

To illustrate this study, we present a chronological tree diagram in figure 1. Although this is the only study we were able to find which provided data on re-randomization in both arms, it likely represents a typical scenario for the assumptions used in our model.

## Results

### Calculations

Assume that *N *patients in a clinical control trial are being randomized (for the first time) in such way that *N*_*C *_patients are randomized into the control group and *N*_*A *_patients are selected into the experimental (alternate) group. In order to simplify our calculations, we have set *N*_*A *_≈ *N*_*C *_≈ *N */ 2. The percentage of people who were re-randomized (randomized for the second time) is *x*. After re-randomization, the total number of events is reported as *E*_*CT *_for the control group and *E*_*AT *_for the experimental group. The total number of events reported is given by *E*_*T *_= *E*_*AT *_+ *E*_*CT*_.

Consider the following two variables:

*E*_*A *_– the number of events in experimental group in the initial randomization;

*E*_*C *_– the number of events in control group in the initial randomization;

These two numbers are the most important in our calculations. We can then estimate the initial risk difference , compare it to the reported numbers and observe the relative error. In the following paragraphs, we will work toward estimating these two variables.

**Figure 2:**
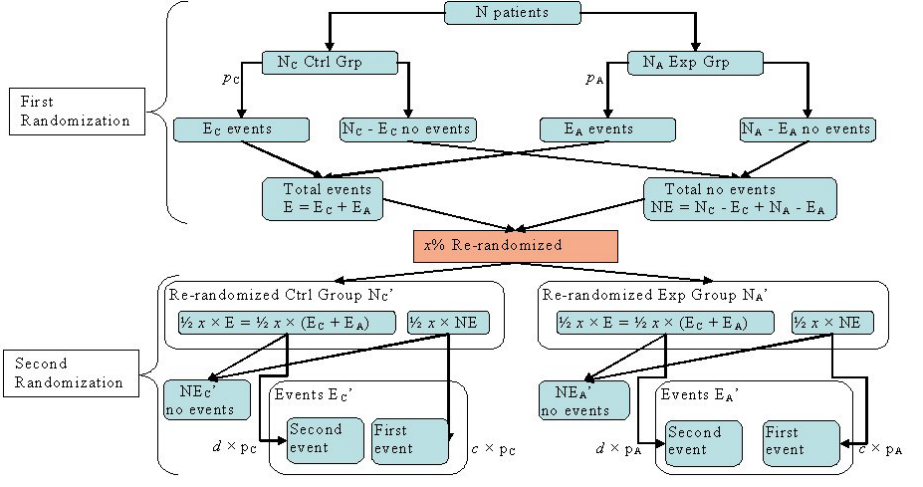
Tree diagram showing the relationships between the variables in the re-randomization process

*E *= *E*_*A *_+ *E*_*C *_– the total number of events in the initial randomization;

*NE *= *N *- *E *– the number of patients without an event in the initial randomization;

 – the risk at which events occurred in the experimental group;

 – the risk at which events occurred in the control group;

 – the risk difference for the initial randomization;

Assuming that re-randomization is approximately evenly divided (if not, we can change the fraction 1/2 with appropriate value), the re-randomized control group and re-randomized experimental group will have the following sizes:

 – the size of experimental re-randomized group;

 – the size of control re-randomized group;

In Figure 2, we summarize the relationship between these variables in a general case. In order to do both, the analysis of difference in outcome between the experimental group and control group, as well as the analysis of repeated randomizations, all of the entries identified in the figure must be determined.

Having in mind that the reoccurrence of an event is *d *times more likely (or less likely if *d *< 1) if the patient comes from the *E *– group (patients with events), and *c *times less likely (or more likely if *c *> 1) if it comes from NE-group (patients without events), and that the events occur at the initial risks *p*_*A *_and *p*_*C *_for the experimental and control groups, respectively, we have:

 – the number of re-randomization events in the experimental group;

 – the number of re-randomization events in the control group;

Therefore, the risk difference of the re-randomization alone, can be calculated as



The overall risk difference for both randomizations, the initial one and the re-randomization, can then be calculated as



or replacing the formula above 

The relative error between the overall risk difference and the risk difference resulting from considering the patients only from the initial randomization is then estimated by the expression



However, the fraction  is usually not reported, and therefore has to be estimated. We will estimate this via the average risk  using the following equations



and



Adding these two equations, we get the quadratic equation in terms of *p *, which can be solved as:

, where *E*_*T *_= *E*_*AT *_+ *E*_*CT*_. Replacing this resulting estimate into the relative difference equation above, leads to



which after some simplifying becomes our main formula:



Therefore, the relative error of the reported risk difference and the actual risk difference before re-randomization can be estimated by the expression depending on *x *– the percentage of the patients who were re-randomized, *d *and *c *– multipliers for the probability of reoccurrence of the event, and the ratio  of the total events reported and the initial number of patients entering the trial.

An Excel file performing all of these calculations and allowing the readers to enter their own data is enclosed as an Additional File 1.

**Figure 3:**
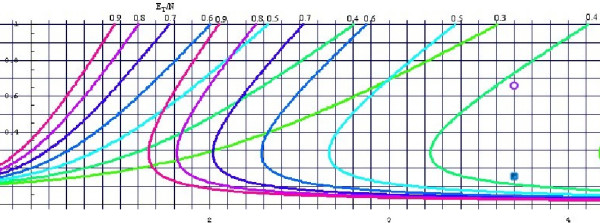
**The 0.05 level curves for the relative difference between the risk difference before and after the re-randomization**. We assume that *c *= 0.40 in this figure. The horizontal axis represents *d *– the multiplier for the probability of reoccurrence of the event after the re-randomization; The vertical axis represents *x *– the percentage of the patients who were re-randomized; The curves represent 0.05 relative error level curves for different values of the ratio  of the total events reported and the initial number of patients entering the trial. The values of the ratio  are indicated at the top of the graph. For a chosen value of the ratio : if a point (*d*, *x*) is between the two level curves with identical value (and color) – the relative error of the risk differences is less than 5%; otherwise – the relative error of the risk differences is more than 5%.

Figure 3 describes our method graphically. The vertical axis is *x *– the percent of the patients who were re-randomized. The horizontal axis is *d *– the multiplier by which the probability of an event changed after an initial occurrence of an event. Figure helps to determine under which circumstances the error in risk difference is less than 5%. The curves represent 5% relative error level curves for different values of the ratio  of the total events reported and the initial number of patients entering the trial. The values of the ratio  are indicated at the top of the graph.

For a chosen value of the ratio : if a point (*d*, *x*) is between the two level curves with identical value – the relative error of the risk differences is less than 5%; otherwise – the relative error of the risk differences is more than 5%.

### Risk ratio

The overall risk ratio after the re-randomization can be defined as , since we assumed that *N*_*A *_≈ (*N */ 2) ≈ *N*_*C *_and . Then, using the definitions of *p*_*A *_and *p*_*C*_, we obtain the following equation:



which is exactly the risk ratio of the initial randomization. Therefore, the overall risk ratio after the re-randomization is equal to the initial risk ratio after the first randomization. Interestingly enough, the risk ratio is not affected by the re-randomization.

### Odds ratio

The overall odds ratio after the re-randomization can be defined as , which can be simplified as 

On the other hand, the odds ratio from the first randomization alone can be expressed as . Using the formulas  and , we get the expression . The relative difference between the odds ratio before the re-randomization and the overall odds ratio can therefore be expressed as



### Data from CSF trials

To illustrate our method, we will use examples from two papers that studied use of CSFs in the treatment of chemotherapy-related febrile neutropenia (FN) and reported the number of patients who were hospitalized and remained in the hospital longer than 10 days [5,6]. In both of these trials some of the patients were re-randomized after first FN episode (hospitalization).

Mitchell et al[5] studied 112 patients in the first randomization for the effect of G-CSF in FN. Seventy-four of these patients were re-randomized (*x *= 66.07%). From a graph in their paper we deduced that 18% and 20% of the patients on GCSF and placebo, respectively, remained in the hospital longer than 10 days. This translates into 17 and 18 events in these two treatment arms . From the graph in the Figure 3, and assuming [4] that *d *= 3.71 and *c *= 0.40, we estimate the relative difference between the reported risk difference evaluated for all the patients, and the risk difference resulting from considering the patients only in the first initial randomization to be only 0.70%. The point corresponding to this example is marked by the purple circle in the Figure 3. In our case, the point is safely between the two identical  level curves (the first and the third curve from the right) indicating that the relative error is smaller than 5%.

Anaissie et al. [6] studied effect of GM-CSF on FN in 92 patients, of which 15.9% were re-randomized (*x *= 0.159). Overall, 18 and 26 patients in GM-CSF and placebo arm remained in the hospital after 10 days . The difference is now 8.23%. The point corresponding to this example is indicated by the blue square in the Figure 3. Note that this time the point is not between two -level curves, indicating that the relative error is larger than 5%.

The odds ratio for this data also changes from *OR *= 0.554 before the re-randomization to the overall value after re-randomization *OR *= 0.535, a change of 3.49%.

### Estimating the risks in the initial randomization

Meta-analysts would also like to know at what risks the events occur in the experimental and control groups before any re-randomization is performed. For example, while in the first example the relative difference between the reported risk difference and the risk difference before the re-randomization was not considerable; in the second example we found a considerable difference.

Some meta-analysts might prefer to use the risks of the events from the initial randomization, i.e., before any re-randomization was performed. In this section we will try to estimate these risks.

By estimating first the average risk  and solving the equations above for *p*, we have , where *E*_*T *_= *E*_*AT *_+ *E*_*CT*_. Using this parameter, the formulas for *p*_*A *_and *p*_*C *_are easily obtained as:



and



Using these estimates, we can get the estimates of *E*_*A *_and *E*_*C *_– the number of events in the initial randomization in experimental and control arms of the trial, respectively.

If the number of patients in each arm of the trial in the initial randomization is given, the events are estimated as *E*_*A *_= *p*_*A*_·*N*_*A *_and *E*_*C *_= *p*_*C*_·*N*_*C*_. If the only number we have is the total number of patients entering the initial randomization, *N*, the events must be estimated more crudely as  and .

In the case of Anaissie et al. example, we obtain the following estimates for the number of events: *E*_*A *_= 14 and *E*_*C *_= 21, and since we are not given the number of patients in each arm, we have to estimate each to 46. Therefore, the numbers we could use for a meta-analysis from this particular trial would be

Experimental     14 / 46

Control     21 / 46

## Discussion

Our method has been developed using the example of febrile neutropenia (FN) in which the patients who once developed FN are considered to be at increased risk for subsequent event [7]. We assume that it is not possible to reliably predict which patients will develop the event of interest from the outset of the trial, but that once the event occurred the risk for subsequent event will be higher in those patients who had developed the first event. Because of this we adopted the values of *d *> 1 and *c *< 1. However, it is conceivable that biology of the process may differ in different circumstances. A meta-analyst is well advised to work with the content-specific experts to address this issue. The same formulas can be used to reflect any combination of the values for *d *and *c *parameters, except when *c *= *d*.

A reader should note that in our model the parameters *c *and *d *work in "conditional probability" framework, i.e., it represents a multiplicative rather than additive approach to asses the effect of previous occurrence of the event on the next one. This makes sense from purely clinical point of view.

### Error analysis

A natural question to ask at this point is "What is the extent of error in the estimation of the parameters *c *and *d *in these formulas, and in particular, in the formula (1.1)."

We first attempted to determine confidence intervals for the parameters *c *and *d*. Unfortunately, since the proportions on top and bottom of the formulas for evaluation of *c *and *d *are obviously dependent random variables, none of the classical examples for evaluation of a confidence interval of a ratio of two proportions can be applied in this case. For example, Sutton et al [8] recommend that a confidence interval for a ratio of two risks  can be calculated using the formula , where . However, this formula, as well as most other formulas, are derived from Taylor's expansion of a transformation of random variables, and depend on the assumption that the ratios *a */ *b *and *c */ *d *are independent (have zero covariance). In our case this assumption can not be applied.

To determine this effect, we used the example of Anaissie et al. [6] and created Table 1.

**Table 1:** Error analysis using Anaissie et al. data

	≈ c											
≈ d		**0.0**	**0.1**	**0.2**	**0.3**	**0.4**	**0.7**	**0.9**	**1.2**	**1.5**	**1.7**	**2.0**
**1.0**		-.286	-.272	-.259	-.245	-.232	-.197	-.162	-.128	-.096	-.064	.033
**1.7**		-.198	-.186	-.175	-.163	-.152	-.122	-.093	-.064	-.036	-.009	.018
**2.4**		-.131	-.121	-.111	-.101	-.091	-.065	-.039	-.014	.011	.035	.059
**3.0**		-.077	-.068	-.059	-.050	-.041	-.018	.005	.028	.050	.072	.093
**3.7**		-.032	-.024	-.016	-.008		.021	.042	.063	.083	.103	.123
**5.6**		.062	.069	.075	.082	.089	.106	.123	.140	.156	.173	.189
**7.5**		.131	.136	.142	.147	.153	.168	.182	.196	.211	.225	.238
**9.4**		.183	.188	.193	.198	.203		.229	.241	.254	.266	.278
**11.2**		.226	.231	.235	.239	.244	.255	.267	.278	.289	.300	.311
**13.1**		.262	.266	.270	.274	.278	.288	.299	.309	.319	.329	.339
**15.0**		.293	.296	.300	.303	.307	.317	.326	.335	.345	.354	.363

The top row of Table 1 represents the estimate of the parameter *c *as it varies from 0 to 2, while the leftmost column indicates value of the estimate of the parameter *d *as it varies from 1 to 15. The numbers in the table show the difference between the real relative difference from formula (1.1) and the relative difference calculated with these alternate values for *c *and *d*. The value .000 (boxed) in the table corresponds to the difference from the RD calculated with *c *= 0.4 and *d *= 3.7 in our example (Anaissie et al. [6]). However, if we assume that *c *= 0.7, and that *d *= 9.4, the value of RD (circled) will be larger by 21.6%, i.e., instead of 8.23%, the value of relative Risk Difference given by the formula (1.1) will be 29.83%. In this case we estimated that the original number of patients who underwent first randomization was 12/46 in experimental group, and 17/46 in the control group, respectively (see above). Note that the while some variation exists, the relative risk difference is not extraordinarily sensitive to the variation in the parameters *c *and *d*.

## Conclusion

In general, it is considered that meta-analyses represent the best method to provide reliable assessment of the effectiveness of health care interventions. However, reliability of meta-analyses depends on the validity of underlying methods used to combine data from the individual studies. One of the key assumptions for the valid MA is the independence of the data being analyzed. As illustrated here, in practice this assumption is often violated. The question for meta-analysts then becomes under which circumstances data can still be properly analyzed even if the independence axiom was violated. In the case of FN, for example, Immunocompromised Host Society issued a guideline in which they considered it acceptable to have multiple randomization of the same patients and reintroduce fully recovered patients into clinical trials [2,9]. However, we believe that the answer regarding the acceptability of re-randomized data is not a simple categorical "yes or no" answer, but it will depend on the amount of error introduced into calculation. If the relative error of risk differences in the results with and without re-randomization is considered small, then such calculations can be safely accepted by meta-analysts. On the other hand, if such an error is considered large, then the analysts should not use the re-randomized data. Instead, the estimates of initial randomization from the method illustrated in the previous section should be used.

It is very intriguing that if the summary effect measure selected is the risk ratio, under the assumptions of our model there is no difference in the final outcome before and after the re-randomization. Therefore, if a meta-analyst is concerned about the effect of re-randomization on the meta-analysis, one way to sidestep the issue and still obtain reliable results is to use risk ratio as the measure of interest.

**Figure 4:**
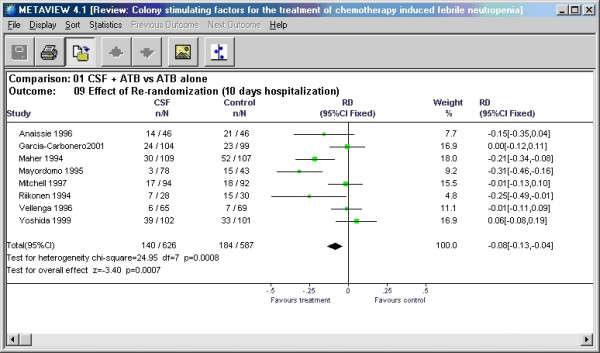
Meta-analysis conducted with Review Manager using the reported data from Anaissie et al. with re-randomized data included.

**Figure 5:**
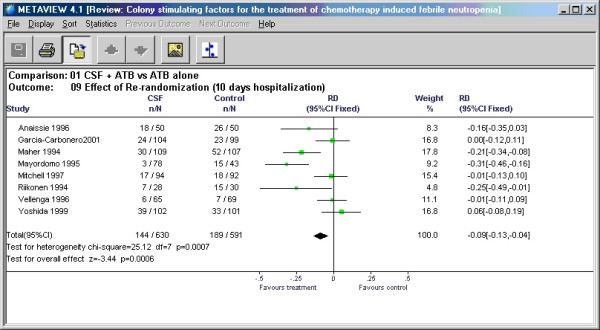
Meta-analysis conducted with Review Manager using our estimates for the data from Anaissie et al. without including the re-randomized data.

In the examples illustrated in this paper, we showed overall risk difference, RD (after second randomization) and RD after first randomization in Mitchell et al. [5] trial differed by relative value of 0.7%. In the case of the trial by Anaissie et al. [6] this difference was 8.23%. It is conceivable that this large relative error (of 8.23%) may affect the results of meta-analysis. Figures 4 and 5 show results of meta-analysis with and without re-randomized data.

In this particular example – change in the input for the meta-analysis did not change the outcome and only marginally changed the pooled risk difference from -9% to -8%. In general, the effect of a given error on the results of meta-analysis can be approximated by multiplying the weight of a given study by the estimate of its relative error. In our example, weight was ~8% and relative error was also ~8% resulting in the change of the summary point estimate in meta-analysis less than 1%. Even when the relative error is high as 29.83% (see above), the summary point estimate would change only by 2.3%. However, should this study have contributed to the meta-analysis with the weight of 40%, the overall change in the summary point estimate would be about 12% (0.4 × 0.3 = 0.12). In general, however, it appears that if the study in question is of small size relative to the rest of the studies included in the meta-analysis, the error introduced by re-randomization will only minimally affect meta-analytic summary point estimate.

Although we developed our method to help with meta-analyses, it should be stressed that our method is also relevant to the analysis of the original trials, and may help assess impact of re-randomization on the results of the trial. We strongly suggest that meta-analysts always compare overall results of their meta-analysis to results based on first randomizations only, as a general control of the reliability of the meta-analysis. In order to make that possible researchers conducting randomized clinical trials would have to report a much more detailed information on their methodology, specifying the numbers of patients being re-randomized, experiencing only one occurrence of an event, experiencing two occurrences of an event, or dropped out of the study.

We believe that the method provided in this paper represents a valuable contribution to the improvement of interpretation of results of clinical trials and in particular their use in meta-analysis. Although the development of this method was stimulated by a concrete problem in the field of febrile neutropenia, practice of re-randomization is used in other areas of medicine such as testing of treatments for epilepsy, recurrent headaches and anti-emetics in cancer chemotherapy.

## Competing interests

The author(s) declare that they have no competing interests.

## Authors' contributions

IH developed most of the formulas for estimation of the risk differences. GHL found the relevant examples and references helping frame the problem in realistic environment. BD and OC conceived of the problem, participated in its framing and coordination, and helped IH to draft the manuscript. All authors read and approved the final manuscript.

## Pre-publication history

The pre-publication history for this paper can be accessed here:



## Supplementary Material

Additional File 1 An Excel file performing all of these calculations and allowing the readers to enter their own data Click here for file
